# Liraglutide and the management of overweight and obesity in people with severe mental illness: qualitative sub-study

**DOI:** 10.1186/s12888-021-03666-5

**Published:** 2022-01-07

**Authors:** Katharine Barnard-Kelly, Clare A. Whicher, Hermione C. Price, Peter Phiri, Shanaya Rathod, Carolyn Asher, Robert C. Peveler, Richard I. G. Holt

**Affiliations:** 1 Southern Health NHS Foundation Trust, Research & Development Department Tom Rudd Unit, Moorgreen Hospital, Botley Rd, West End, Southampton SO30 3JB UK; 2 BHR Limited, Fareham, Hampshire, UK; 3 Academic Department of Psychiatry, College Keep, Terminus Terrace, Southampton, SO14 3DT UK; 4 Southampton National Institute for Health Research Biomedical Research Centre, University Hospital Southampton NHS Foundation Trust, Southampton, UK; 5 Human Development and Health Academic Unit, Faculty of Medicine, University of Southampton, Tremona Road, Southampton, SO16 6YD UK

**Keywords:** Liraglutide, Obesity, Overweight, Severe mental illness, Schizophrenia, Psychosis

## Abstract

**Background:**

People with severe mental illness are two to three times more likely to be overweight or have obesity than the general population and this is associated with significant morbidity and premature mortality. Liraglutide 3 mg is a once daily injectable GLP-1 receptor agonist that is licensed for the treatment of obesity in the general population and has the potential to be used in people with severe mental illness.

**Aims:**

To record the expectations and experiences of people with schizophrenia, schizoaffective disorders or first episode psychosis taking daily liraglutide 3 mg injections in a clinical trial for the treatment of obesity.

To seek the views of healthcare professionals about the feasibility of delivering the intervention in routine care.

**Methods:**

Qualitative interviews were undertaken with a purposive sub-sample of people with schizophrenia, schizoaffective disorders or first episode psychosis with overweight or obesity who were treated with a daily injection of liraglutide 3 mg in a double-blinded, randomised controlled pilot study evaluating the use of liraglutide for the treatment of obesity. Interviews were also conducted with healthcare professionals.

**Results:**

Seventeen patient participants were interviewed. Sixteen took part in the baseline interview, eight completed both baseline and follow-up interviews, and one took part in follow-up interview only. Mean interview duration was thirteen minutes (range 5-37 min). Despite reservations by some participants about the injections before the study, most of those who completed the trial reported no challenges in the timing of or administering the injections. Key themes included despondency regarding prior medication associated weight gain, quality of life impact of weight loss, practical aspects of participation including materials received and clinic attendance. Healthcare professionals reported challenges with recruitment, however, overall it was a positive experience for them and for participants.

**Conclusion:**

Liraglutide appears to be an acceptable therapy for obesity in this population with limited side effects. The quality of life benefits realised by several intervention participants reinforce the biomedical benefits of achieved weight loss.

## Background

Over the last thirty years, the prevalence of obesity in the general population has increased dramatically, with individuals with severe mental illness, such as schizophrenia or bipolar disorder, being disproportionately affected. The rates of overweight and obesity are 2-3-fold higher in people with severe mental illness than in the general population [1]. Obesity adversely affects the physical health and psychological well-being of people with severe mental illness and contributes to the 2-3 fold excess morbidity and mortality experienced by this population. If weight gain is attributed to treatment, this can lead to treatment discontinuation and risk of relapse of psychosis.

Short-term lifestyle interventions has been shown to have some efficacy in supporting weight reduction in people with severe mental illness however a recent meta-analysis by Speyer et al. reported such lifestyle interventions have limited impact with only reduced mean BMI by 0.63 kg/m^2^, equivalent to a weight loss of 2.2 kg [2]. The challenges of implementing lifestyle change in people with severe mental illness are well-known and there has been a lack of long-term effectiveness. As such, it is important to find alternative approaches to manage overweight and obesity in this population. Several pharmacological treatments have been subject to clinical studies, but currently the only drug therapy licensed for the treatment of antipsychotic-associated weight gain, or for obesity in people with severe mental illness is orlistat [3]. The long-term use of this drug, however, is extremely limited due to high discontinuation rates, thus it is of limited value in routine clinical practice [4].

Glucagon-like peptide-1 (GLP-1) receptor agonists are a class of drugs that have been used to treat type 2 diabetes for 15 years. These were mostly administered by injection, with a frequency ranging from twice daily to once weekly, although a daily oral preparation has recently been launched. As well as effectively lowering glucose and HbA_1c_, their use is associated with weight loss.

The observed weight loss seen in people with diabetes treated with GLP-1 receptor agonists led to clinical trials investigating their use as treatments for obesity. To date, only one GLP-1 receptor agonist, liraglutide, is licensed for this indication, with a higher dose of 3.0 mg daily than the maximum 1.8 mg daily used for diabetes. Others, however, are in development. Prior to our study, there have been three completed trials of GLP-1 receptor agonists in people with severe mental illness, two using exenatide and one using liraglutide [5–7]. These trials results in a mean 3.71 kg greater weight loss after 16 weeks in the active treatment arms compared with placebo. These studies, however, used the doses used to treat diabetes. We therefore undertook a pilot feasibility to compare the use of liraglutide (maximum dose 3.0 mg daily) with placebo in obese or overweight people with schizophrenia, schizoaffective disorder or first episode psychosis [8, 9]. This study demonstrated that although recruitment was challenging, once participants were enrolled, retention and adherence to the trial medication was similar to previous studies of liraglutide 3 mg daily in the general population. Furthermore, those randomised to liraglutide treatment experienced clinically relevant weight loss, which was consistent with previous trials. The aim of this sub-study was to record the expectations and experiences of participants taking daily liraglutide injections during the pilot trial as well as the views of healthcare professionals about the feasibility of delivering the intervention in routine care.

## Research design and methods

### Design

This qualitative study was a sub-study of a double blind randomised controlled pilot trial undertaken between 24 July 2018 and 5 May 2020 in community and in-patient mental health centres and primary care in Southern Health NHS Foundation Trust, UK. The protocol for the study and main results have been published previously [8,9].

The aim of this sub-study was to record the expectations and experiences of people with schizophrenia, schizoaffective disorders or first episode psychosis taking daily liraglutide 3 mg injections in a clinical trial for the treatment of obesity and to seek the views of healthcare professionals about the feasibility of delivering the intervention in routine care.

In short, 47 participants (aged 21-64 years) with a clinical diagnosis of schizophrenia, schizoaffective disorder or first episode psychosis and treated with an antipsychotic medication were recruited to the study. Their body mass index ranged from 29.4 – 59.7 kg/m^2^. After a baseline assessment, participants were randomised in a 1:1 ratio to treatment with liraglutide 3.0 mg by daily injection or a matched placebo. All participants, carers, and study personnel except the pharmacy team were blinded to treatment assignment. The liraglutide was used according to the current EU licence for Saxenda®; the starting dose was 0.6 mg per day, which was titrated every week to a maximum of 3.0 mg per day. The duration of treatment was 6 months. Participants were taught how to self-inject and text reminders were sent to support self-administration.

### Participants

All 24 participants in the liraglutide arm of the main trial were invited to take part in the qualitative interviews. Of these, seven participants did not consent to be contacted. Interviews continued until no new topics were raised. In addition, two healthcare professionals delivering the intervention were interviewed.

### Qualitative interviews

In-depth semi-structured interviews were conducted with participants at baseline and at completion of the intervention phase. Questions explored expectations and experience of taking part in the trial, as well as broader experiences of attempted weight loss. Interview scripts were developed in collaboration with potential participants and piloted prior to use in the study to ensure relevance and acceptability of questions.

Two qualitative researchers undertook content and thematic analyses [10]. A thematic analytical approach was used in which transcripts were cross-compared to understand and identify patterns and experiences that cut across different people’s accounts and the underlying reasons for similarities and differences in their experiences and views. Key aspects of the analysis included exploring: (a) participants’ perceptions, experiences and behaviours related to therapy use; (b) participants’ overall perceptions of the therapy; (c) cross-comparison between perceptions and experiences as well as identifiable factors related to differences and similarities. Interviews were audio-recorded and then transcribed verbatim before being reviewed for accuracy, de-identified and analysed.. A coding framework was developed to capture key themes and each coded theme was subjected to further analyses to identify subthemes and illustrative verbatim quotes. A qualitative expert in collaboration with research team established the codebook using open coding. All data was independently reviewed and analysed by both researchers with regular meetings to discuss potential disagreements in coding. Inter-rater reliability between coders was measured. Agreement was reached by consensus.

### Ethics

The study was conducted in keeping with Good Clinical Practice (GCP) and the International Conference of Harmonisation (ICH) standards. South Central - Hampshire B Research Ethics Committee (REC) approved the study on the 17 April 2018 (Reference: 18/SC/0085). Southern Health NHS Foundation Trust sponsored the study (SHT325). The trial was registered with the WHO Primary Registries Universal Trial Number (UTN) is U1111-1203-0068 and the European Clinical Trials Database (EudraCT) number: 2017-004064-35. Participants were provided with oral and written information about the study and gave written informed consent.

## Results

### Intervention participants

Seventeen of the 24 intervention arm participants were interviewed. Of these, 16 took part in baseline interview, 9 completed both baseline and follow-up interview and one took part in follow-up interview only. Mean interview duration was thirteen minutes (range 5-37 min). Despite reservations by some participants about the injections before the study, most of those who completed the trial reported no challenges in timing of or administering the injections. Table 1 shows the responses from baseline qualitative interviews while Table 2 shows the expectations and prior experiences of participants coming into the study. Table 3 shows the responses from follow-up qualitative interviews.

**Table 1:** Responses from baseline interview

Question	Yes	No
Have you tried to lose weight before taking part in the study?	13	3
Is there anything you are particularly optimistic about? - The potential to lose weight (*n* = 9)	9	2
Is there anything you are particularly concerned or worried about?	9	7
- Injecting (*n* = 6)		
- Side effects (*n* = 3)		
Concerns about side effects?	3	12
- Vomiting (*n* = 1)		
- Diarrhoea (*n* = 1)		
- Unsure (n = 1)		
Safety concerns?	1	14
- Needle bending (n = 1)		
Do you expect any challenges in timing of doses?	1	8
- Find it hard to stick to things (n = 1)		
Impact of timing on routine?	1	9
- Need to take pen to work (n = 1)		

Numbers do not always total 16. Responses reflect unique codes, some participants gave more than one response; some participants either did not know or did not answer some questions

**Table 2:** Responses to Specific Questions in the baseline interview

Question	Responses	Frequency
**First, please tell me why you chose to take part in this study?**	Put on lots of weight/am overweight	8
	- Due to medication	5
	- Due to hospital stays	1
	Offered to take part/it was recommended by a healthcare professional	6
	To lose weight	5
	To benefit other people and me in the future	4
	Give it a go/why not	2
**Have you tried to lose weight before taking part in the study? If yes could you tell me a little bit about that experience?**	Altered diet	9
	- Due to joining a commercial weight loss programme	4
	Increase in exercise	5
	Healthcare professional recommended it	4
	It was working	4
	Commercial weight loss programmes too expensive to keep up a membership	2
**What are your expectations going into the study?**	It was explained well, I know what to expect	4
	No expectations	3
**Is there anything you are particularly optimistic about?**	The potential to lose weight	7
	I’ve already started, it’s going OK	2
**Is there anything you are particularly concerned or worried about?**	I’m not worried	6
	Injecting	5
	Side effects	3
	- Such as vomiting	1
	- Such as diarrhoea	1
**Any concerns about side effects?**	I’m not worried	9
**Do you expect any challenges in timing of doses?**	Same time everyday	2
**Impact on routine?**	Same time everyday	4
**Practicalities?**	Manageable	1
**Anything else?**	Can I expect a difference in my mental health?	2
	Information all clear, no concerns	2

Numbers do not always add up to 16. Responses reflect unique codes and some participants gave more than one response or did not express an answer some questions

**Table 3:** Responses from follow-up interview

Question	Yes	No
Did you lose any weight?	5	4
Were your expectations met?	2	2
Anything unexpected?	2	7
Change in diet or exercise?	6	3
- Trying to keep to smaller portions (*n* = 4)		
- Eating healthier (*n* = 4)		
Did you feel safe when taking liraglutide?	7	2
Was there anything you were particularly concerned or worried about?	1	4
- The size of the needle (*n* = 1)		
Any side effects?	8	5
- Sickness (*n* = 2)		
- Diarrhoea (*n* = 2)		
- Constipation (*n* = 2)		
- Extreme stomach pain (*n* = 2)		
Any additional stress?	3	2
- Stressed about travel not being in my control (*n* = 1)		
- Due to side effects (*n* = 2)		
Did taking liraglutide impact on your everyday living or daily routine in terms of additional burden or benefit?	3	6
- Side effects were a burden (n = 3)		
Did you experience any challenges in timing of doses?	4	5
- Forgetting (*n* = 3)		
- Fitting injections around work (n = 1)		
If a similar clinical trial were to be conducted, would you recommend it to a friend if they met the inclusion criteria?	9	0

Numbers do not always add up to 9. Responses reflect unique codes and some participants gave more than one response or did not express an answer for some questions

### Key themes

#### Medication associated weight gain

Several participants reported considerable weight gain associated with the antipsychotic medications they are taking. For example,

- ‘I went to the gym but there’s no point trying. It’s a waste of time. Then I get less motivated, I’m not very motivated as it is, so it was really hard. Like fighting an uphill battle’ [001].
- ‘Ever since being diagnosed with mental health, all sorts of medications made me put on weight … I’m not happy with my weight’ [002]
- ‘I need to lose weight … it was dreadful .. my medication doesn’t help’ [012]
- ‘I’ve been going [to Slimming World] for 18 months … it worked for a while but the medications I’m on make me hungry’ [013]
- ‘I’m fat … I’ve had a problem with my weight for 4-5 years, it’s linked to my medication’ [015]

For some participants, this was associated with reduced motivation and despondency as illustrated by the following verbatim:

- ‘I went to weightwatchers and it didn’t work … so many different diets!’ [010]
- ‘I’ve tried not eating .. eating healthy … exercise … I just can’t lose it’ [016]

#### Impact of study on quality of life

Several participants commented that there had been an improvement in their quality of life, which they attributed to the medication, including a positive impact on family relationships. Some participants reported that their quality of life was improved directly as a result of the weight loss. A participant who dropped two clothes sizes described their experience in the study as “life-changing” as they were feeling both physically and mentally better.

- ‘I can walk properly again with improved mobility … I have better breathing … my mood is improved’ [021]
- ‘It’s been life-changing, a brilliant experience. Before, I was in crippling pain and couldn’t walk even short distances so was isolated further and further. Now I can walk to the corner shop. I’d do it all again tomorrow’ [024]
- I felt better when my clothes were getting looser, it kind of put me on a bit of a buzz …. My daughter noticed it, mentioned it quite a bit .. she could put her arms around for a cuddle. When I was bigger, she couldn’t as her hands couldn’t touch but now she can which is nice’ [007]
- ‘I dropped two clothes sizes, I’m feeling better physically and mentally’ [024]

In the next two examples, the respondents acknowledge the impact on quality of life and family noticing changes.

- ‘My family says I’m nicer to be around … mentally I’m in a better place …. My mum’s stopped nagging about food and acting like the food police’ [021]
- ‘My family noticed my weight loss … told me I’m looking good again. I’m up and about more, engaging with life more, not drowsy all day, wasn’t sleeping all day, it’s a big big help, it’s lovely’ [021]

#### Study information and support from trial was well-received

A common theme from participants was the trial support. On the whole, participants felt well supported (*n* = 7) by the staff who were described to have explained the information really well.

- ‘I felt supported, I didn’t feel abandoned at all’ [026]
- ‘Information and advice were brilliant, I knew what to expect… I always had five minutes to ask any questions’ [021]

Furthermore, participants said that the information sheets were good and they found them useful (*n* = 8).

- ‘The information sheet was quite concise, it was pretty good. No jargon’ [013]
- ‘It was explained really well .. I felt I got a lot of support, felt very supported’ [003]

Text messages were a key theme of trial support highlighted by participants (*n* = 6). Three participants reported issues with forgetting to take their injections on occasion, but the text messages were a useful reminder. Although not every participant needed the reminders, they were still useful; for example, the following participants said,

- “the messages set routine”,[020]
- “the text messages everyday helped because they get unsure if they have taken something or not” [013].

One participant who did not need the reminders still described them as a “good back up” plan [026].

Whilst all participants interviewed reported being pleased that they had participated in the study, it is clear that those who did not lose any weight were disappointed. Each of these participants assumed they were in the control group, i.e. receiving the placebo. Despite this disappointment, they did not regret their participation.

#### Practical aspects of clinic attendance

Despite describing clinic attendance as satisfactory (*n* = 3), two participants found the journey to appointments problematic because of the additional stress of not being in control over timing while waiting for their taxi. One participant suggested booking the taxi 20-30 min earlier to reduce this burden.

Three participants were concerned about potential side effects of vomiting and diarrhoea before the study started. On completion of the trial, side effects of constipation, diarrhoea, vomiting and stomach pain were each reported by two participants.

#### Healthcare professional perspective

Two healthcare professionals, who were responsible for delivery of the pilot study, including recruitment, intervention delivery supervision and follow-up, were interviewed. Whilst neither were involved in the protocol development, both were involved from the start of the pilot study. Both reported recruitment to be challenging but believed the intervention was feasible if delivered as part of routine care. A particularly positive and rewarding experience was the dramatic weight loss experienced by some participants and the resulting positive impact on their quality of life. The nature of drug delivery, i.e. injection therapy, was seen as a downside by one healthcare professional who felt this was a factor affecting recruitment and a stressor for some participants. No safety concerns were identified by either healthcare professional, with no concerns regarding timing or doses, impact on daily living or adherence to the treatment regimen.

## Discussion

Seventeen patient participants took part in the interviews. This represents almost half of participants in the LOSE Weight pilot study and the majority of the intervention participants. Participants were representative of the overall study participants in both arms, as well as those who completed or withdrew from the study.

Weight loss can be challenging, particularly in this population due to the obesogenic nature of medications and often sedentary lifestyles [1]. Unsurprisingly, therefore, many participants reported having become despondent with their previous weight loss attempts and were increasingly less motivated to keep trying to lose weight. Evidence shows that almost half of appointments for mental healthcare users are missed [10]. The most common reasons for missed appointments included forgetting, work commitments, no transportation and financial constraints. Despite this, the participants in the current study had a high rate of attendance at study appointments, with healthcare professionals commenting on the commitment to attend. It is possible that the attendance was associated with travel reimbursement, however, none of the participants reported reimbursement to have been a factor in their attendance. Practical issues around the timing of transport were reported by a minority of participants, however, these did not deter full involvement in the study.

The injectable nature of drug delivery was perceived by healthcare professionals to be a barrier to recruitment. One suggested that having had an injection pen available for potential participants to see and hold when approached about the trial could have improved recruitment. A common misconception was that the injections would be similar to depot injections, with a much larger needle. This was, understandably, off-putting for many. Participants reported being pleasantly surprised by the injection device and only one participant expressed concern regarding injections, namely that the needle may bend. They did not experience such an event. The results show that participants did not find the injections to be burdensome and easily accommodated them within their usual daily routine.

Text message reminders in healthcare services have received much attention [11]. A systematic literature review reports that text messages appear to be an effective reminder to improve appointment attendance and medical engagement [11]. Such messages have demonstrated benefits and these benefits are clearly experienced by the current population. Text reminders in the current study were reported to be useful and reassuring by participants, although not always necessary. The text messages were not seen as a nuisance by any of the participants.

It is crucial to balance the demands of additional medication with potential benefit and it is encouraging that the quality of life benefits associated with successful weight loss were considerable and life-changing for some participants.

### Strengths and limitations

Key strengths of the current study include conducting interviews both at baseline and follow-up, with a broad range of participants taking part at both or either time points. This enabled the capture of a broader range of views. Similarly, conducting interviews with healthcare professionals delivering the study provided valuable data on feasibility of delivery of the intervention in routine clinical care and the experiences of delivering as well as receiving the intervention.

The study was limited by challenges in recruitment and the views of participants may not be generalisable to all participants in the study or those who declined to participate. It is not possible to say whether data saturation was reached. The consequences of weight loss or not were very personal and extremely impactful for some participants, both physically and emotionally. Whilst there were key themes with several participants reporting similar things, the inclusion of saturation in this context might be misconstrued that future participants would have nothing new to add, which is unlikely to be the case.

## Conclusion

The quality of life benefits realised by several intervention participants reinforce the biomedical benefits of achieved weight loss. Believing they were in the placebo group was very disappointing for several control participants and future research should consider whether a cross-over design may improve recruitment, retention and participant satisfaction.

## Data Availability

The datasets used and/or analysed during the current study available from the corresponding author on reasonable request.

## Abbreviations

kg: Kilograms
CI: Confidence Intervals
HbA_1c_: Haemoglobin A_1c_
N: Number

## References

[1] Kendler KS, Gallagher TJ, Abelson JM (1996). The National Comorbidity Survey: lifetime prevalence, demographic risk factors, and diagnostic validity of nonaffective psychosis as assessed in a US community sample. Arch Gen Psychiatry.

[2] Speyer H, Jakobsen AS, Westergaard C, Norgaard HCB, Pisinger C (2019). Lifestyle Intervedntion for weight Management in People with serious mental illness: a systematic review with Meta-analyses, trial sequential analyses, and Meta-regression analyses exploring the mediators and moderators of treatment effects. Psychother Psychosom.

[3] Mizuno Y, ST, Nakagawa A, Yoshida K, Mimura M, Fleischhacker WW, Uchida H (2014). Pharmacological strategies to counteract antipsychotic-induced weight gain and metabolic adverse effects in schizophrenia: a systematic review and meta-analysis. Schizophr Bull.

[4] Citrome L, PR, Kezouh A, Levine M, Etminan M (2007). Long-term persistence with orlistat and sibutramine in a population-based cohort. Int J Obes.

[5] Ishøy PL (2017). Effect of GLP-1 receptor agonist treatment on body weight in obese antipsychotic-treated patients with schizophrenia: a randomized, placebo-controlled trial. Diabetes Obes Metab.

[6] Siskind DJ, Russell AW, Gamble C, Winckel K, Mayfield K (2018). Treatment of clozapine-associated obesity and diabetes with exenatide in adults with schizophrenia: a randomized controlled trial (CODEX). Diabetes Obes Metab..

[7] Larsen JR (2017). Effect of Liraglutide treatment on prediabetes and overweight or obesity in clozapine- or olanzapine-treated patients with schizophrenia Spectrum disorder: a randomized clinical trial. JAMA Psychiatry.

[8] Whicher CA, Price HC, Phiri P, Rathod S, Barnard-Kelly K, Reidy C, et al. Liraglutide and the management of overweight and obesity in people with schizophrenia, schizoaffective disorder and first-episode psychosis: protocol for a pilot trial. Trials. Open access 20 November 2019 https://trialsjournal.biomedcentral.com/articles/10.1186/s13063-019-3689-5. 10.1186/s13063-019-3689-5 PMC6868690 31747930

[9] Whicher CA, Price HC, Phiri P, Rathod S, Barnard-Kelly K, Ngianga K, et al. The use of liraglutide 3.0mg daily in the management of overweight and obesity in people with schizophrenia, schozoaffective disorder and first episode psychosis: results of a pilot randomised double-blind placebo-controlled trial. Diabetes Obesity Metab. 2021; In press. 10.1111/dom.14334 33528914

[10] Morse JM, Field PA (1995). Qualitative research methods for health professionals.

[11] Ramlucken L, Sibiya MN (2018). Frequency and reasons for missed appointments of outpatient mental health care users in the uMgungundlovu district. Curationis.

